# Biomarker development for immuno-oncology and cancer immunotherapy: simultaneous digital counting of nucleic-acids and proteins at 800-plex

**DOI:** 10.1186/2051-1426-3-S2-P81

**Published:** 2015-11-04

**Authors:** Alessandra Cesano, Joseph M Beechem

**Affiliations:** 1 NanoString Technologies, Seattle, WA, USA

## Background

The ability of mutated cells to give rise to pathological cancer relies upon the capability of these cells to interact with the immune system and ultimately evade immune recognition and suppress immune activity through multiple (differently expressed) mechanisms. Although our understanding of the tumor-host interaction has dramatically increased in the last ten years, greatly accelerating the pace of immunotherapy drug development, a clear need remains for clinical biomarkers capable of informing on patient selection, biology-based combination therapy, and adverse effects monitoring. Due to the highly regulated, multi-step, multi-tissue, multi-compartmental cancer-immunity cycle, it is unlikely that measurements limited to one or few analytes (e.g., PD-L1) – or even single analyte classes (e.g., DNA, mRNA, or protein) – will be informative enough for predictive/prognostic immunotherapy applications. New developments in multiple biomarker analyte-class optical barcode counting significantly reduce this problem.

## Methods

Recent work from the Weissleder-lab [1] has shown how optical barcode technology can be utilized for multiplexed digital counting of proteins, and be combined with simultaneous digital counting of nucleic-acids on a single nCounter analysis system. NanoString has expanded upon this original work and developed a cancer immune-profiling technology that simultaneously measures 770 mRNA's (unique signatures for 24 infiltrating immune cell types plus extensive immune-signaling pathways) plus 30 key immuno-oncology proteins (including PD-1, PD-L1, PD-L2, CTLA4, OX40) using small amounts of clinically relevant samples (~ 50,000 PBMCs for mRNA+protein, 1 or 2, 5um slices for mRNA alone). This technology (“RNA:Protein”) is forming the basis for multi-year collaborations between NanoString and both MD Anderson (Houston TX) and the Cancer Immunotherapy Trials Network (CITN) to discover unique multi-analyte-type (mRNA + protein) biomarker signatures to guide cancer immunotherapy.

## Results

NanoString gene expression profiling has also been recently highlighted [2] as a method to select for patients that will benefit from anti-PD1 based therapy (Keytruda) in a number of different cancer types. This technology is also being expanded to work on multi-analyte detection completely from archival FFPE slices. Several examples of the utilization of RNA:Protein immune-profiling technology to develop biomarker signatures relevant to immuno-oncology will be presented.
